# Barriers to and enablers of the implementation of an ICF-based intake tool in clinical otology and audiology practice—A qualitative pre-implementation study

**DOI:** 10.1371/journal.pone.0208797

**Published:** 2018-12-11

**Authors:** Lisette M. van Leeuwen, Marieke Pronk, Paul Merkus, S. Theo Goverts, Johannes R. Anema, Sophia E. Kramer

**Affiliations:** 1 Amsterdam UMC, Vrije Universiteit Amsterdam, Otolaryngology-Head and Neck Surgery, Ear & Hearing, Amsterdam Public Health, De Boelelaan, Amsterdam, Netherlands; 2 Amsterdam UMC, Vrije Universiteit Amsterdam, Public and Occupational Health, Amsterdam Public Health, De Boelelaan, Amsterdam, Netherlands; University of Queensland, AUSTRALIA

## Abstract

The authors are developing an intake tool based on the Brief International Classification of Functioning Disability and Health Core Set for Hearing Loss, by operationalizing its categories into a Patient Reported Outcome Measure. This study was aimed at identifying enablers and barriers to using this tool as perceived by hearing health professionals (HHPs) and patients. Focus groups and interviews were held with HHPs (ENT surgeons, N = 14; audiologists, N = 8) and patients (N = 18). Interview questions were based on the Capability-Opportunity-Motivation-Behavior (COM-B) model. Using the COM-B model and the Theoretical Domains Framework (TDF), transcript fragments were divided into meaning units, which were then categorized into capability-, opportunity- and motivation-related barriers and enablers. These were further specified into TDF domains. HHP barriers included: lack of time to use the tool (O); and fear of being made responsible for addressing any emerging problems, which may be outside the expertise of the HHP (M). Enablers included integration of the tool in the electronic patient record (O); opportunity for the patient to be better prepared for the intake visit (M); and provision of a complete picture of the patient’s functioning via the tool (M). Patient’ barriers included fear of losing personal contact with the HHP (M); and fear that use of the tool might negatively affect conversations with the HHP (M). Enablers included knowledge on the aim and relevance of the tool (C); expected better self-preparation (M); and a more focused intake (M). These findings suggest that an intervention is needed to enhance HHPs’ knowledge, skills and motivation regarding the relevance and the clinical usefulness of the tool. Providing clear and specific information on the purpose of the tool can also enhance patient motivation. For both HHPs and patients, opportunities relating to the (digital) administration and the design of the tool provide additional targets for successful implementation.

## Introduction

Adults with ear and hearing problems may experience both physical impairments and psychosocial consequences that can significantly impact their functioning in daily life [1]. *Functioning* is a multidimensional construct. According to the World Health Organization (WHO) it reflects the interplay of an individual’s body structures and functions, activities, participation and contextual factors. In other words, a whole-person perspective is required to assess functioning of an individual with a particular health condition (here: ear/hearing problems) [2]. It is acknowledged that ear and hearing health care should consider a patient’s total functioning to provide optimal care and obtain optimal outcomes (e.g., [3–5]). However, ear and hearing problems are often understood in the context of the specific disease (medical perspective) with a focus on relieving the impairments that exist on the level of body functions and body structures. Such an approach does not include the level of participation (restrictions) and the individual’s personal and environmental context, and therefore only partially describes and addresses the consequences of ear and hearing problems [4, 6]. Any inclusion of these aspects in current clinical practice is unlikely to be standardized.

The use of WHO’s International Classification of Disability and Health (ICF, [2]) as a frame of reference to assess an individual’s total functioning may facilitate a better understanding of the (consequences of) ear or hearing problems for the individual patient and improve health care provision (e.g., [3, 6, 7]). However, the ICF consists of more than 1400 categories, which is not workable in clinical practice, and ICF Core Sets have therefore been developed. These are lists of selected categories that have been demonstrated to be the most relevant for describing the functioning of a person with a specific health condition. Following the need for a standard instrument to facilitate a common validated way for assessing the effect of hearing loss on the lives of adults, the ICF Core Sets for Hearing Loss (CSHL) were established according to strict procedures prescribed by the World Health Organization [8]. The Core Sets were developed based on a series of preparatory studies which included the researcher, clinician and patient perspectives and an international consensus process [4]. The Comprehensive CSHL contains 117 categories to be taken into account in a multi-professional comprehensive assessment of a patient’s functioning with hearing problems. The Brief CSHL includes 27 of the 117 categories and represents the minimal set that should be assessed in a person with HL in single discipline encounters or clinical trials.

In a prior study, we examined the content validity of the CSHL with respect to the intake procedures used for patients in Dutch oto-audiology practices [9]. Results revealed some gaps in the current intake documentation and indicated that implementation of the CSHL in the Dutch practice could complement current practice and help professionals obtain an integral perspective of the patient’s functioning [9]. However, a drawback of the CSHL is that they define ‘what to measure’, but not ‘how to measure’. Additional steps are therefore required to enable the use of the CSHL in clinical practice, i.e., i) operationalization of the CSHL into a practical intake tool and ii) implementation of this instrument in clinical practice. In a parallel study we focus on step i) by operationalizing the Brief CSHL into a Patient Reported Outcome Measure (PROM) (results will be described elsewhere). The current study focuses on step ii). Throughout the study we used a rough conceptual description of the intake tool which was presented to the study participants. In the remainder of this paper we will refer to this conceptual description as the ‘ICF-based intake tool’.

It is often argued that PROMs can facilitate patient-centered care. However, simply implementing a PROM does not imply patient-centered practice, unless it serves to strengthen the patient-clinician relationship, promotes communication about things that matter to the patient, increases patients’ knowledge about their health, and facilitates their involvement in their own care [10]. It is therefore important to realize that the intake tool itself does not represent patient-centered care, but it may be a step towards it. The profile generated by the intake tool can be used as a starting point in the intake, to facilitate communication between patients and clinicians and foster an equal partnership in determining treatment. The degree of patient-centeredness is the result of this process. In a next step, guidelines and pointers for the clinicians on how to discuss the intake tool’s outcomes and the patient’s treatment options in a patient-centered way will therefore be required. However, as a first step, the context and mechanisms through which the intake tool is meant to affect change should be considered [11]. Testing for the presence of factors that are necessary to influence and produce desired outcomes (here: using the intake tool in a way so it facilitates patient-centered care) is therefore important [12]. This involves the careful examination of implementation context/processes that support or impede the utilization of the intake tool [11–13]. This study is a critical evaluation of the implementation of the ICF-based intake tool from the perspectives of the hearing health professional (HHP) and the patient. This is important, given that if the newly developed ICF-based intake tool is poorly implemented and not routinely used as intended, the potential benefits will not be achieved.

The importance of careful implementation is reflected in the fact that although there is mounting evidence that PROMs can impact upon processes of care and clinical outcomes [12, 14–18], this impact may vary widely [19–21]. Implementation of PROMs in clinical practice implies shifts in practices for both patients and health care providers in order to accommodate the collection and the feedback of the PROM information. Changing these practices is known to be a challenging process [22–24]. Potentially impeding or enabling factors for the implementation of PROMs can be found at various levels, and include factors related to the PROM itself (e.g., simplicity and adaptability to the context), professionals and patients involved (e.g., knowledge and expectations), and the social, organizational, economic and political context (e.g., costs-effectiveness) [25]. Trying to implement a change in clinical practice requires consideration of individual *behavior change* of all parties involved [26–28]. Successful adoption of a new practice or intervention (here: use of an ICF-based intake tool) is enhanced when it is compatible with the users’ values and current needs [22, 23]. It is therefore important for the implementation of the ICF-based intake tool in oto-audiological clinical practice to understand the specific information HHPs find useful in their setting and the obstacles they perceive to the routine assessment of PROMs as part of clinical care. Patient engagement in using PROMs is of paramount importance to limit the impact of response burden and to enhance successful PROM implementation [13, 29]. In other fields of healthcare, reported barriers to the use of PROMs include: lack of time, preference for physiological measures (in contrast to self-report measures), perceived lack of clinical relevance, uncertainty in interpreting PROM result and patient burden [30]. Currently no information is available about the barriers and enablers related to the implementation of PROMs in the clinical oto-audiology setting. A better understanding of the perceived enablers of- and barriers to the use of an ICF-based intake tool, and subsequent targeting of these enablers and barriers could contribute to successful implementation and routine use of the ICF based intake tool in clinical practice.

Implementation researchers strongly recommend the use of a theoretical framework to increase the likelihood of identifying and subsequently targeting the full range of enablers and barriers to implementation (e.g., [31]). This study therefore used a theory-based approach to identify barriers to and enablers of the use of the ICF-based intake tool perceived by HHPs and patients. HHPs included ENT surgeons and post-academically skilled medical physicist audiologists (further referred to as audiologists). The careful identification and categorization of barriers and enablers, which is described in the current study, is necessary to develop an intervention for the implementation of the intake tool (e.g., [31]), which will be determined in a future study.

## Materials and methods

### Study design

A qualitative study was performed using structured individual interviews with patients and HHPs (one audiologist), and semi-structured focus groups with HHPs (i.e., ENT surgeons and audiologists). The focus groups and individual interviews were performed to identify the possible enablers of and barriers to the use of the ICF-based intake tool, and to identify what changes implementation of the tool would require in current practice.

### Description of the ICF-based intake tool

In the current study, the patients and HHPs were introduced to the ICF-based intake tool and its intended use in clinical practice. We provided the following information:

- The patient was to be asked to complete a questionnaire assessing relevant aspects of their functioning prior to his/her intake visit;
- The patient’s responses were to be made available to both the patient and the HHP and thereby serve as a communication tool that could guide the intake and subsequent treatment process.

The overall aim of the overarching research project is to improve patient-centered care in otology and audiology. Hearing impairment is a condition central to each of these disciplines and multiple disciplines are often involved in the tertiary care. To enable better coordination and continuity of care we therefore opted for an integrated approach to collect functioning information in the initial contact across all patients, independent of the specific oto/audiology discipline through which the patient enters the care system. Note that the CSHL was developed for adults with hearing loss, which explains why we created an ICF-based intake tool for patients who come to an Audiology Center and/or an ENT practice for hearing loss-related complaints. ENT practices in the Netherlands serve patients with a wide range of ear complaints, of which hearing loss is the most prominent one which often coexists with ear disorders. Also, an exact diagnosis often is yet to be determined at the start of a rehabilitation trajectory. In addition, for the same hearing complaint, patients can come into the hospital via a referral to either an ENT surgeon or the audiologist. These factors underline the need for a common language and reference system that functions across professional boundaries. This should start immediately after patients are referred to our hospital. Based on these facts, and given our preference for a uniform tool, we have chosen to create this new intake tool using the CSHL as reference.

### The theory-based approach

A large pool of psychological theories explaining behavior change are available to guide implementation research. Examples are the health belief model (HBM; [32]), theory of planned behavior (TPB; [33]), and the transtheoretical model (TTM; [34]). However, literature suggests that such models may fail to consistently and reliably explain variability in human behaviors (e.g., [35]). Moreover, many of these theories use overlapping constructs and lack guidance for selecting the best one [36]. The Capability Opportunity Motivation-Behavior (COM-B) model was developed by integrating concepts from 19 frameworks of behavior change identified in a systematic review by Michie and colleagues (2011) [28] and has been applied successfully by others in the context of hearing health care [37, 38].

The model proposes that for someone to engage in a particular behavior (B) they must be physically and psychologically able (C), have the social and physical opportunity (O) to perform the behavior and, lastly, be motivated (M) to perform the behavior. Motivation covers automatic processes such as emotional reactions and impulses and reflective processes such as intention and beliefs.

In addition to the COM-B model we used the Theoretical Domains Framework (TDF) for a more detailed evaluation of HHP and patient barriers and enablers. The TDF is an integrated theoretical framework synthesized from 128 theoretical constructs from 33 theories judged most relevant to implementation questions [39, 40]. It has been linked to the COM-B model by Michie and colleagues (2014). Based on the TDF, the C, O, and M components of the COM-B are further divided into 14 key theoretical domains of behavior that an implementation intervention might focus on [39]. The TDF provides a more granular understanding of psychological capability and reflective motivational processes than the COM-B alone [40]. Moreover, the TDF was recommended in a recent paper to guide the development of behavior change interventions for clinicians and patients aimed at addressing the barriers to and maximizing the enablers of PROM implementation [24]. The domains of the TDF include:

- knowledge;
- cognitive and interpersonal skills;
- memory, attention and decision processes;
- behavioral regulation;
- environmental context and resources;
- social influences;
- social/ professional role and identity;
- beliefs about capabilities;
- optimism;
- intentions;
- goals;
- beliefs about consequences;
- reinforcement;
- emotions.

A specification of each COM component and its related TDF domain(s) is provided in S1 Appendix.

### Recruitment and sampling

#### HHPs

HHPs were included via convenience methods. An invitation e-mail was sent from the research team to a staff member of each setting. We then provided staff with further information and contacted them to arrange the focus groups. The focus groups were organized in or following existing (educational) meetings. Aiming at a sample representative of Dutch clinical hearing health care settings we included ENT surgeons and audiologists from the Amsterdam UMC, location VUmc, department of Otolaryngology-Head and Neck Surgery, a secondary Otolaryngology Department and a secondary Audiological Center in the study. These included:

- Ten ENT surgeons at the section of Otology of the department of Otolaryngology- Head and Neck Surgery of Amsterdam UMC, location VUmc (tertiary setting).
- Four ENT surgeons at the department of Otolaryngology of the WestFriesGasthuis (WFG) hospital in Hoorn (secondary setting).
- Seven audiologists at the University Audiology Center of the department of Otolaryngology- Head and Neck Surgery of Amsterdam UMC, location VUmc (tertiary setting);
- One audiologist at the Audiology Center Holland Noord (ACHN) in Alkmaar (secondary setting).

Table 1 shows the characteristics of the participating HHPs.

**Table 1:** Characteristics of the Hearing Health Professionals (N = 20) participating in the study.

Variable	ENT surgeons		Audiologists	
Setting*	1	2	3	4
**Number of participants**	8	4	7	1
- *ENT residents/ audiologists in training*	6	-	2	-
**Gender** *male/female*	6/2	3/1	5/2	1/0
**Age** *mean; SD*	31.6; 5.2	50; 5.7	42.3; 8.1	52
**Years of work experience** *mean (range)*	5.9 (2–15)	16.3 (11–26)	8.3 (1–20)	24

*Setting: Amsterdam UMC, location VUmc section Otology = 1, WFG = 2, Amsterdam UMC, location VUmc Audiology Center = 3, ACHN = 4

ENT = ear nose and throat

#### Patients

Patients with ear and/or hearing problems were recruited at Amsterdam UMC, location VUmc in Amsterdam. Only patients meeting the following inclusion criteria were invited: visiting the outpatient clinic of the Amsterdam UMC, location VUmc for the first time, above the age of 18 years. A maximum variation strategy was applied with regard to the ear/hearing problem and age, in order to form a heterogeneous group covering the full spectrum of otology/audiology patients characteristics. Recruitment of participants took place in the waiting room via the HHPs assistants. The HHPs assistants selected eligible patients, announced the study, and asked whether patients would potentially be willing to participate. LvL then explained the study more in detail and invited the patient to participate in an interview. Patients were scheduled for an interview prior to their visit to the outpatient clinic for an intake with an audiologist or ENT surgeon. Eighteen patients were included and were asked to sign informed consent forms. Table 2 shows their sociodemographic and condition-related characteristics. All patients were interviewed by LvL at the outpatient clinic of Amsterdam UMC, location VUmc, in a separate room before their scheduled intake consult.

**Table 2:** Characteristics of the patients (N = 18) participating in the study.

Variable	Total	Otology patients	Audiology patients
**Number of participants**	18	12	6
**Gender** *male/female*	55.6	58.3	33.3
**Age** *mean (range)*	54.5 (18–84)	52.6 (18–77)	58 (20–84)
**Country of birth** *The Netherlands/Other country*	16/2	10/2	6/0
**Otology diagnosis** *(%)*			
• *Diseases of external ear (ICD-10*: *H60-H62)*	-	2	Na
• *Diseases of middle ear and mastoid (ICD-10*: *H65-H75)*	-	3	Na
• *Diseases of inner ear (ICD-10*: *H90-H95)*	-	2	Na
• *Other diseases of the ear (ICD-10*: *H90-H95)*	-	5	6
**Audiology diagnosis group** *(%)*			
• *Tinnitus*	-	Na	1
• *Hearing loss*	-	Na	3
• *Cochlear Implant*	-	Na	2

Na = not applicable; ICD-10 = 10^th^ revision of International Classification of Diseases and Related Health Problems

### Focus groups and interview procedures

The interview guides that were used for the structured group discussions and individual interviews are shown in S2 Appendix. The topics and questions were designed to identify barriers and enablers based on the components of the COM-B model.

#### Focus groups with HHPs

One discipline-specific focus group meeting was conducted within each setting. One audiologist was interviewed individually. A topic list was used to facilitate group discussion. The topics related to current practice and perceptions regarding using the ICF based intake tool in routine care. HHPs were asked about their current practice and what they thought could be potential tools or methods to support their intake (Q1-Q3), and what requirements they had in mind in order for them to actually use such a tool in their clinical practice (Q4-Q6). The focus groups and individual interview with the audiologist of ACHN took about one hour.

#### Individual interviews with patients

A structured interview guide was used in the patient interviews. Patients were asked about their experiences with intakes, (Q1), and what they considered important in intakes (Q2-Q5). Secondly, the ICF-based intake tool was introduced and its intended use was briefly explained. Patients were asked what they thought of filling out and using such a tool for the intake (Q6). Subsequently, they were asked to indicate which conditions the ICF-based intake tool would have to meet for them to use it (Q7-Q10). Interviews took between 15–30 minutes.

LvL conducted all the interviews and moderated the focus groups. Because of the large number of HHPs participating in the two focus groups at Amsterdam UMC, location VUmc, an observant was present during these focus groups to help monitor the group process.

### Analysis

All focus group meetings and interviews were audio recorded. LvL transcribed and anonymized all interviews. Data analysis (content areas; i.e., explicit areas of relevant content) was based on the qualitative data analysis method by Graneheim and Lundman, as described in Knudsen et al. (2012) [41]. Data saturation was reached for the patients when all patient groups commonly seen in oto-audiology practice were represented in the sample and the final interview yielded no new unique responses. Due to time restrictions, data collection among the HHPs was not based on data saturation and significant barriers and enablers may therefore have been missed. The interview transcript fragments that were relevant to the content areas were divided into meaning units. Subsequently, the COM-B model and the TDF were used to categorize the meaning units into capability-, opportunity- and motivation-related barriers and enablers, and further specified into TDF domains.

In order to ensure reliability of the analysis procedure, [41], one randomly selected patient interview transcript and 10% of two randomly selected focus group transcripts were independently analyzed by MP and SEK. The percent agreement between the analyses of LvL, MP, and SEK was calculated for the categorization of the meaning units into COM-B components as well as TDF domains. Any discrepancies were discussed until consensus was reached on the optimal categorization.

### Ethics

The study was approved by the Medical Ethical Committee of the Amsterdam UMC, location VUmc, Amsterdam; the Netherlands. Data collection was carried out between November 2016 and February 2017.

## Results

The barriers and enablers found for HHPs and patients are presented in Tables 3 and 4, and Tables 5 and 6, respectively. The identified factors, together with illustrative quotes from participants, are described in more depth below and are categorized according to the COM-B components. Results are presented separately for HHPs and patients. Supplemental text is provided in brackets when further clarification was deemed necessary for the readability of quotes.

**Table 3:** Identified barriers to the use of the ICF-based intake tool as perceived by hearing health professionals.

COM-component	Setting*	Theme	TDF
Psychological capability	1,2	*In current practice*, *assessing/discussing psychosocial and contextual factors are not included (or to a limited extent)*	Skills, Behavioral regulation
	1,2	*In current practice*, *HHPs lack the skills to address patients’ psychosocial complaints*	Skills
	1	*Lack of knowledge regarding internal referral pathways for patient problems relating to psychosocial factors*	Knowledge
Physical opportunity	1,2,3,4	*Reviewing a patient’s responses on a long intake tool and acting on them is not perceived as feasible in daily practice*	Environmental context and resources
	1,3	*High turnover practice limiting the time for the intake*	Environmental context and resources
	1,3	*Limited time frame per intake*, *restricting the number of topics to discuss*	Environmental context and resources
	1,2	*Perceived (extra) time investment to use the intake tool*	Environmental context and resources
	2,4	*Open-ended questions should be avoided*, *as they take too much time to review and are difficult to address*	Environmental context and resources
Reflective motivation	1,2,3	*Feeling responsible for addressing any functioning topics that arise from the intake tool*	Social/ professional role and identity
	1,2	*Perception that the intake tool is suitable in an Audiological Center or as an research tool*, *but is less suitable in the Otology intake practice*	Social/ professional role and identity
	1,2	*Attitude that it is sufficient to rely on own questions and own structure during the intake and consultation as compared to a pre-defined and structured format*	Beliefs about capabilities
	1,2,3	*Not feeling competent in using the intake tool with regard to having to address patient problems in areas beyond the HHP’s expertise or capabilities*	Beliefs about capabilities
	1	*Uncertainty about the patient’s satisfaction with the provided care*	Optimism (pessimism)
	1	*Uncertainty about whether patients will perceive the intake tool as relevant or not*	Optimism (pessimism)
	1	*Perception that the benefit to patient care is only theoretical*, *and does not work in practice*	Optimism (pessimism)
	1,2,3	*Perception that the intake tool will not affect the choice of a treatment strategy (for most patients)*	Goals
	1,2	*Attitude that the psychosocial factors included in the intake tool do not match the expertise and interest of the HHP*	Goals
	1	*Perception that there is no need for a comprehensive list of questions*, *as there are only a few questions that have to be asked to the patient to know what is going on*	Intentions
	1,2,3	*Perception that the intake tool has no added value in patients with well-defined ear/hearing problems (e*.*g*., *ear infection*, *presbyacusis) for whom the treatment options are evident in their view (e*.*g*., *medication*, *hearing aids)*	Beliefs about consequences
	1,2,3	*Fear that additional questions in the intake tool (as compared with current practice) will raise additional problems the HHP has to assess*	Beliefs about consequences
	1,2,3	*Fear that patients expect that the intake tool is used and topics are discussed*, *while the HHPs may not consider it relevant*: *mismatch between expectations of the patients and the HHP’s actions*	Beliefs about consequences
	1,3	*Fear of getting responsibility for addressing problems that would arise not be in the expertise of the HHP*	Beliefs about consequences
	1,2	*Concern that use of the intake tool would create more work*	Beliefs about consequences
	3	*Fear that questions will only be asked because these are part of the intake tool*, *and not because these are relevant to the patient*	Beliefs about consequences
	1,3	*Concern that the cause of the problems reported by patients may not always be ear/hearing-related*	Beliefs about consequences
	2	*Fear that format of the tool could cause a fixed frame for the intake consultation*, *without room for nuance*	Beliefs about consequences

*Setting: 1 = Amsterdam UMC, location VUmc section Otology, 2 = WFG, 3 = Amsterdam UMC, location VUmc Audiology Clinic, 4 = ACHN

COM-B = Capability, Opportunity, Motivation- Behavior; TDF = Theoretical Domains Framework; HHP = hearing health professional

**Table 4:** Identified enablers of the use of the ICF-based intake tool as perceived by hearing health professionals.

COM-component	Setting*	Theme	TDF
Psychological capability	1,2,3,4	*Knowledge that psychosocial factors may affect the daily functioning of the patient and can underlie the patient’s ear and hearing complaints*	Knowledge
	3,4	*In current practice discussing psychosocial- and contextual factors are already part of the intake practice*	Skills, Behavioral regulation
	3,4	*In current practice various structured questionnaires/intake-forms are already part of the intake*	Skills, Behavioral regulation
Physical opportunity	1,2,3,4	*Patient’s responses should be presented in a simple overview that is easy to use*	Environmental context and resources
	1,2,3,4	*The intake tool should be provided in a digital format*	Environmental context and resources
	1,2,3,4	*The intake tool should be self-administered by the patient*	Environmental context and resources
	1,2,3,4	*The intake tool should predominantly contain closed-ended questions*	Environmental context and resources
	1,2,3,4	*The intake tool should be integrated into the hospital/center’s electronic system*	Environmental context and resources
	1,2	*The overview of patient’s responses should only show the problem areas of the patient’s functioning*, *and should not include factors the patient reports no problems with*	Environmental context and resources
	2,4	*Use of the intake tool should be easy regarding accessibility*, *language*, *and administration method*	Environmental context and resources
	1,4	*The intake tool should include prompts and triggers for treatment options or referral pathways to direct the actions of the HHP*	Environmental context and resources
	3,4	*The intake tool should be designed as a decision tree format*	Environmental context and resources
Reflective motivation	3	*Sense of professional responsibility to address all the complaints and problems of the patient (including complaints not relating to the ear and hearing domain)*	Professional role and identity
	1,2,3	*Perceived added value of using the intake tool in patients with complex problems (i*.*e*., *patients referred from tertiary care*, *and patients with tinnitus or vertigo)*	Intentions
	3,4	*Ambition to get the (more) complete picture of the patient’s functioning*	Intentions
	2	*Willingness to try out the intake tool*	Intentions
	3	*Recognition that a complete picture of the patient cannot be guaranteed in current practice and that this may be facilitated by using the intake tool*	Goals
	1	*Perceived value of the intake tool*, *if the tool ensures increased patient satisfaction with the care provided*	Goals
	1	*Use of an efficient intake tool may save time*	Goals
	2	*Perceived value of the intake tool facilitating a better preparation by the patient*	Goals
	3	*Perceived value of the intake tool in managing the patient’s expectations regarding treatment/intervention outcomes (e*.*g*., *to indicate the areas where the patient may or may not expect improvement)*	Goals

*Setting: 1 = Amsterdam UMC, location VUmc section Otology, 2 = WFG, 3 = Amsterdam UMC, location VUmc Audiology Clinic, 4 = ACHN

COM-B = Capability, Opportunity, Motivation- Behavior; TDF = Theoretical Domains Framework; HHP = hearing health professional

**Table 5:** Identified barriers to the use of the ICF-based intake tool as perceived by patients.

COM-component	Theme	TDF
Psychological capability	*Information and instructions of the intake tool should not be too long or extended*	Skills
	*Use of medical jargon should be avoided*	Skills
Physical opportunity	*The time that is needed to complete the questionnaire should not be too long*	Environmental context and resources
Motivation	*The questions in the questionnaire should not be duplicated in the intake conversation*, *i*.*e*., *the patient’s responses should be reviewed by the HHP before the intake visit*	Goals
	*The intake should not be shortened or dominated by the intake tool*	Goals
	*Providing the patient’s responses to the HHP prior to the intake visit should not negatively affect or replace the (open) conversation with the HHP*	Beliefs about consequences
	*Use of the intake tool should not reduce the personal attention of the HHP for the patient and his/her problems*.	Beliefs about consequences

COM-B = Capability, Opportunity, Motivation- Behavior; TDF = Theoretical Domains Framework; HHP = hearing health professional

**Table 6:** Identified enablers of the use of the ICF-based intake tool as perceived by patients.

COM-component	Theme	TDF	
Psychological capability	*Information on the aim and relevance of the intake tool are important*	Knowledge	
	*Instructions on how to fill out and use the intake tool are important*	Knowledge/ skills	
Physical opportunity	*Preference to complete the intake tool at home (versus in the clinic)*	Environmental context and resources	
	*Opportunity to fill out the intake tool both on paper and digitally (on the computer)*	Environmental context and resources	
	*In case of a digital intake tool*: *easy and straightforward accessibility is important (i*.*e*., *log-in process)*	Environmental context and resources	
	*In case of a digital intake tool*: *a possibility to save the responses after completion of the questionnaire is important*, *to be able to use it in preparation for the intake with the HHP*	Environmental context and resources	
Social opportunity	*Perceived value of social support from family members with filling out the intake tool*	Social influences	
Motivation	*Motivation to use the intake tool would be strengthened when the information on the purpose of the intake tool is clear*	Intentions	
	*Motivation to use the intake tool would be strengthened if patients could start with reporting their problem/ reason for visit*, *so that their specific complaint or need is placed at the center of the intake*		Goals
	*Perceived value of collecting all relevant information regarding one’s functioning*, *and sharing this information with the HHP before the intake*		Goals
	*Perceived value of the intake tool facilitating better preparation of oneself for the intake visit*		Goals
	*Perceived value of the intake tool contributing to a better understanding or insight into the impact of one’s own ear/hearing problem*		Goals
	*Perceived value of the intake tool contributing to better care provision*		Goals
	*Perceived value in sharing results to help future patients*, *science*, *and/or society*		Goals
	*Perceived time-efficiency in the intake*		Beliefs about consequences
	*Perceived value of the intake tool in directing the intake towards the actual complaints and needs of the patient*		Beliefs about consequences
	*Perceived value of the intake tool in facilitating the intake conversation*, *because both the patient and health professional are prepared better*		Beliefs about consequences

COM-B = Capability, Opportunity, Motivation- Behavior; TDF = Theoretical Domains Framework; HHP = hearing health professional

### Barriers and enablers perceived by HHPs (Tables 3 and 4)

#### Capability

Both ENT surgeons and audiologists expressed that psychosocial factors (which would be captured by the ICF-based intake tool) can influence the daily life functioning of patients with ear and hearing problems. This was identified as a *psychosocial capability* (COM-B) by HHPs that can be further linked to the TDF category *knowledge*. HHPs knowledge on the relevance of the ICF is therefore a factor that could act as a potential enabler to implementing the intake tool.

*“These are factors [the bio-psychosocial factors in the intake tool] that affect the well-being of the patient and may also direct the patient’s complaints*.*”* (ENT surgeon)

Another *psychological capability* that was identified as an enabler, was that audiologists reported that discussing psychosocial- and contextual factors with their patients is a common part of their current intake practice. This factor was linked to the *skills* and *behavioral regulation* category of the TDF because it covers clinical experience.

*“One question I ask very often is ‘in which situations do you notice your problems specifically in your daily life*, *in what aspects of your daily life do you encounter them*?*’”* (audiologist)

*“We ask what their personal environment looks like*, *what kind of people what kind of situations*, *and what role the problem plays here in*.*”* (audiologist)

Another factor that was identified as an enabler linked to *skills* and *behavioral regulation* (TDF) was the audiologists’ familiarity with using structured intake-forms and questionnaires. In contrast to audiologists, the ENT surgeons reported that they are not used to assessing and discussing psychosocial and contextual factors with their patients in current practice, which was therefore identified as a barrier for this group. Also, they expressed their concerns about their current lack of *skills* to deal with patients’ psychosocial complaints. Addressing such complaints was perceived to be outside their area of expertise.

*“We are not specifically trained for that [to address psychosocial factors]*.*”* (ENT surgeon)

Furthermore, some ENT surgeons indicated a lack of *knowledge* (TDF) regarding the hospital’s internal psychosocial referral pathways, and did not appear to know about the social worker as a member of the multidisciplinary audiology team in the department of Otolaryngology- Head and Neck Surgery in the Amsterdam UMC, location VUmc.

#### Opportunity

Identified opportunity barriers and enablers all related to the physical environment (COM-B level) and were categorized under *environmental context and resources* (TDF level). Both ENT surgeons and audiologists perceived the limited time available to use the intake tool as an important potential barrier. Specifically, reported concerns related to the short time frame per intake and the high turnover practice that was already pressuring current usual practice. Use of the tool was viewed as adding even more to their task load, as yet another extensive list of topics had to be reviewed and addressed. It was viewed as unworkable in daily practice.

*“We only have ten minutes for the intake*, *and in those ten minutes the patient needs to come in*, *you need to do the intake conversation*, *the physical examination*, *and explain the treatment*. *Everything that makes the intake more complex or broader will be frustrating*, *I think”*. (ENT surgeon)

*Environmental context and resources* (TDF) related to enablers were also raised. Both ENT surgeons and audiologists indicated that a potentially workable method is to ask the patient to complete the tool independently, before the intake, and preferably online. They expressed a preference for closed-ended rather than open-ended questions to prevent overly exhaustive descriptions of complaints by patients. This would make the complaints time-consuming to review and difficult to address. In addition, ENT surgeons indicated their preference for “*an easy overview of the results”* in which “*it is immediately clear what is filled in by the patient*”, emphasizing that this overview should be very simple and easy to use. ENT surgeons suggested that this overview should only show the problem areas of the patient’s functioning, and should not include factors the patient reported no problems with. In this way the HHP could immediately focus on the real problem areas during the intake.

ENT surgeons reported that prompts and triggers for appropriate treatment options or referral pathways to other appropriate health professionals–corresponding to the fields of functioning that would pop up as ‘problem areas’–could work as a potential enabler of using the tool.

“*It could be useful if we had referral trajectories within the hospital [*..*] that you get a pop-up saying ‘refer to discipline x’ and that this discipline is located in room y*.*”* (ENT surgeon)

Another enabler mentioned by both ENT surgeons and audiologists was that the overview of the patient’s functioning should be integrated in the hospital/center’s electronic system. Moreover, HHPs reported that the intake tool must be accessible to patients, including quick and easy access to the digital intake tool before the intake visit, use of simple language (i.e., suitable for low literate patients), and flexibility with regard to administration method (e.g., availability via desktop, laptop, smartphone, but also on paper in case the patient does not use a personal computer).

#### Motivation

All identified motivational barriers and enablers were linked to the component *reflective motivation* (COM-B level). Both ENT surgeons and audiologists expressed concerns about being responsible for addressing any problems reported by the patients in the intake tool (TDF: *professional roles and responsibilities*), specifically problems that in their opinion are not directly related to the patient’s ear/hearing problems and/or to their own expertise or capabilities (e.g., depressive complaints) (TDF: *beliefs about their capabilities*). They mentioned they may not want to focus on problem areas that they cannot treat. This barrier was linked to the TDF domain *goals* of the intake.

“*You want to know about those factors that you can actively intervene on*. *So you want to ask those questions that provide information about what to do with the patient*. *The factors we cannot intervene*, *I do not want to focus on*.*”* (audiologist)

ENT surgeons feared that including such items in the tool might lead patients to expect that they would address these problems (TDF: *beliefs about consequences*); and if this did not happen there could be a mismatch between patient expectations and the HHP’s actions.

“*If patients report that they are really depressed and you only address the factors relating to the ear*, *because that is what matters to you [as a doctor]*, *you do not match the expectation you created by the questions you asked*.*”* (ENT surgeon)

In the audiologists’ focus group, the opposite opinion also emerged: it is their professional responsibility to address all the complaints and problems of the patient, even if they are only indirectly related to the ear or hearing problem. It was mentioned that these complaints should be addressed at least to the degree of checking whether the patient is already being seen by another health care practitioner.

Some of the ENT surgeons and audiologists questioned whether the tool was relevant for all patient groups (TDF: *beliefs about consequences*). They did not see added value of the tool for patients with what they viewed as ‘well-defined ear/hearing problems’, for example patients with simple ear infections or patients with typical presbyacusis and, in their view, evident treatment options (e.g., medication and hearing aids, respectively). In this light, it was also questioned whether additional information on psychosocial and contextual factors would change treatment strategy.

“*A large proportion of otology problems are concrete problems*. *For example*, *presbycusis and ear infections are problems I do not need all sorts of lists for in advance*.*”* (ENT surgeon)

“*If you split a patient’s complaint and needs up into all sorts of categories during your anamnesis and you end up with a hearing aid yes or no either way*, *the question is whether it is useful to know about all the patient’s complaints*.*”* (audiologist)

Also, it was believed that a lot of (psycho-social) problems are already solved by the standard treatment strategy, so there is no need to address the psychosocial problem separately; TDF: *intentions* to use the intake tool.

“I*f a patient has a running ear*, *which you identify in 3 questions and by a quick look into the ear*, *you prescribe eardrops*. *And if the person has been feeling miserable and depressed because of the running ear*, *then it will not change the treatment strategy you have chosen*.*”* (ENT surgeon)

Another shared concern relating to *beliefs about the negative consequences* of the intake tool (TDF) was that when you list all potential problems that patients with ear/hearing problems may have, patients will be more likely to report them and you end up with a list of problems patients might have not raised without such a list. Other *beliefs about the negative consequences* of the tool (TDF) included audiologists’ concern that a standardized tool might lead to an overly automated intake process. This could compromise open conversation with and attention to the patient and implied the risk of the intake tool replacing the patient-HHP interaction. They also were concerned that questions would be asked only because they are listed in the intake tool, and not because they are relevant for all individual patients.

“A *disadvantage could be that you feel you have to ask the question*, *because it is on the list*, *while you normal would not have asked this particular patient*.*”* (audiologist)

Similarly, an ENT surgeon expressed his fear that use of the tool would cause a “*fixed frame without room for nuance*”.

Regarding the suitability of the intake tool into clinical practice, ENT surgeons did perceive added value in its use in the Audiological Center (for audiology patients). For the ENT intake practice (otology patients) it was generally regarded as unsuitable. The ENT surgeons perceived the AC as (already) being more focused on the rehabilitation of psychosocial aspects of hearing problems. Another suitable application of the tool was seen in scientific research, as an instrument for measuring pre- and post- intervention outcomes. These perceptions were categorized as a barrier related to ENT surgeons’ *professional role and responsibility* (TDF).

An important enabler mentioned by the audiologists was that they strive for a comprehensive review of each individual patient, to improve their current practice to get the complete picture of the patient’s functioning with his or her problems (TDF: *goal*).

“*A fundamental issue is that if I try to get a complete picture of a patient from the referral letter*, *the anamnesis and the audiogram*, *am I overlooking anything*? *I may think that I can build a complete picture from those reports*, *but there may be another factor that is not mentioned in these documents*. *So if you have an instrument that can guarantee that completeness*, *that would be good*.*”* (audiologist)

Some audiologists saw the added value of the intake tool in managing the patient’s expectations regarding treatment (TDF: *goal*): the patients’ responses can be used to indicate the areas where patients cannot expect improvement.

“*That you are able [with the tool] to prepare the patient that it [the intervention or treatment] will give improvement in some areas*, *and not in some other areas*.*”* (audiologist)

A motivational enabler mentioned by ENT surgeons that was categorized under positive *beliefs about the consequences* of the intake tool (TDF) was the perceived added value of the tool for the intake, including a better preparation by the patient. This added value was mainly seen for patients with complex problems, e.g., patients with tinnitus and vertigo.

Another factor that would enhance the motivation of ENT surgeons to use the intake tool is if the tool ensured increased patient satisfaction with the care provided (TDF: *goal*). However, they expressed their concern that the benefit to patient care is only theoretical, and not practical in clinical practice (TDF: *pessimism*).

“*The medical problem tells you where you can help the patient*, *and that is quite limited*. *If you look at very broad domains of functioning*, *then it's only a small part of what we are able to treat*. *[*..*] Again*, *the politically correct answer would be everything to improve patient's wellbeing*, *but that is theory; in practice your options are limited”*. (ENT surgeon)

Another mentioned motivational enabler relating to the *goal* of the intake (TDF) was the tool’s potential to make the intake process more time-efficient. However, because the required investment in time and effort to use the tool was viewed as greater than current practice, it was not considered a realistic option for most of their patient groups.

### Barriers and enablers perceived by patients (Tables 5 and 6)

#### Capability

Most patients expressed that they needed clear information on the aim and the relevance of the intake tool to use it. These factors were identified as domains of *knowledge* (TDF) enabling patients to use the tool. In addition, patients mentioned that it was important to have clear instructions on how to fill out the questionnaire and interpret and use the intake tool’s output, in order to facilitate discussion of their responses with the HHP (linked to TDF domains *knowledge* and *skills*).

“*It is important to explain why you should fill in the questions and how it can help in the conversation [with the HHP]”*. (patient)

However, it was also indicated that such an explanation should be short and concise. Instructions on how to respond to particular questions were only appreciated if essential for the correct interpretation of the question. Furthermore, patients underlined that medical jargon should be avoided (TDF: *knowledge*).

#### Opportunity

Similar to the HHP reports, identified enablers in the *physical opportunity* component (COM-B level) mainly related to the TDF domain *environmental context and availability of resources*. A number of patients indicated their preference for a digital intake tool to be completed at home. They felt they had more time and tranquility there to complete the questionnaire at their convenience. The reported maximum time considered adequate for completing the intake tool was about 15 minutes, although reactions ranged from *“Definitely not too long*, *5 to 10 minutes”* to *“As long as necessary*, *maybe an hour*?*”*. Most patients indicated that they would like to receive their responses after completion of the questionnaire, but found it difficult to say in what format. Most patients indicated that a printed version, and the ability to save it as a PDF with all questions and answers listed would be sufficient. One patient indicated that access to the tool should be easy and straightforward. The log in process should be designed accordingly.

“*If you have to login into a questionnaire*, *it may not always be as expected so you cannot log in*. *If people run into a roadblock here*, *including myself*, *I would find that very annoying*. *So it must be something easy*, *not something complicated*.*”* (patient)

At the COM-B level *social opportunity*, support from immediate family members with filling out the intake tool was identified as a potential enabler by younger adults (ages 18 and 19) (i.e., support from parents) and older adults (i.e., support from the partner, children or a caregiver). These patients expressed that they would value the opportunity to discuss the questions and answers with their family members.

“*Interviewer: Do you want or need support from others*, *like your partner or caregiver*, *to fill in the intake tool*? *Patient*: *Yes maybe some questions I would like to discuss with her [patient referring to patient’s mom]*.*”* (patient)

#### Motivation

All identified motivational barriers and enablers were linked to the component *reflective motivation* (COM-B level). A potential barrier related to the TDF domain *beliefs about the negative consequences* of the intake tool was the fear that the intake tool would get in the way of the (open) conversation with and personal attention from the HHP. Some patients therefore indicated that the use of the intake tool should not negatively affect or replace the conversation with the HHP. Also important was that the intake tool should not shorten or dominate the intake.

Information on the purpose of the questionnaire and what would subsequently be done with the patient’s responses were identified as important enablers for the patient’s *intention* to use the intake tool.

“*It must be clear what will happen with it [the responses that the patient has provided]*, *and what the purpose is*, *that must be clear too*.*”* (patient)

Generally, patients seemed to value the idea of collecting all relevant information regarding their functioning, and that this information was shared with the HHP before the intake took place. They perceived various potential benefits (TDF: *beliefs about the positive consequences* of the intake tool), including better preparation by both the patient and the HHP. It was regarded as important that the HHP would actually use the intake tool and not duplicate questions in the face-to-face intake.

“*If you let me fill in a questionnaire in advance*, *you [the HHP] should let me know that you have read it [*..*] But do not ask the questions again*, *or show that you [the HHP] did not read it*. *Because then I will feel like I am not heard*.*”* (patient)

Regarding other *beliefs about the positive consequences* of the intake tool (TDF), patients valued the possibility of being able to prepare for the intake by filling out the questionnaire beforehand at home. Some patients indicated being quite nervous during intakes, which often made them forget to ask the questions they intended to ask.

"*[..] when I am there [in the consulting room]*, *you are often put on a very different track*, *so you forget your own questions [*..*] There's always some nervousness that makes you forget what you intended to ask*.*"* (patient)

Some patients mentioned that the intake tool could help them order/structure their thoughts. The overview of their responses would help them during the intake to address their concerns and questions. Some patients also valued the perceived effect of facilitating more depth and more focus on their specific complaints during the intake, because many questions have already been asked and answered.

"*You may get a better conversation with the doctor because you stay away from the standardized facts that usually take up a large amount of the time of the intake conversation*, *and now those facts are already there*. *Then you can go into more depth*.. *yes if it [the tool] has such a function*, *then I am all for it*.*"* (patient)

“If you *have the opportunity to fill in a questionnaire or to make comments in advance*, *and formulate your own ideas about what may be causing your complaints*, *you can be much more focused during the intake conversation with the doctor*. *I think that is very important*, *or could be anyway*.*”* (patient)

Related to this, providing information beforehand was perceived to be potentially time-efficient in the intake (TDF: *goals*). Another motivational enabler to fill in the intake tool was that it could contribute to being heard and taken seriously by the HHP (TDF: *beliefs about the positive consequences*).

“*I would be motivated to use the intake tool if I think it helps to be taken seriously and therefore to receive better care*.*”* (patient)

Some patients indicated that they would be motivated to fill out the questionnaire if they could start with reporting their problem or the reason for their visit. Subsequently, the questions in the different functioning-categories could follow. In this way, their specific complaint or needs would be placed at the center of the intake. This method was categorized as a *goal* of using the intake tool (TDF). Some patients mentioned their motivation to complete the intake tool and share their results to help future patients, science, and/or society (TDF: *goals*). They mentioned hoping that the factors generated by the intake tool would provide insights for the development of new treatment options.

#### Overlap in barriers and enablers perceived by HHPs and patients

There is some overlap between several barriers and enablers mentioned by HHPs and patients. Regarding capability, for example, both HHPs and patients indicated the need to enhance their knowledge on and skills for using the intake tool. Regarding the opportunity-related factors HHPs and patients indicated that the time needed to complete the questionnaire and review the results respectively, should be limited. In addition, HHPs and most patients preferred a digital tool that is easily accessible for patients. As to motivational factors, both HHPs and patients expressed their concern about the intake tool negatively affecting the intake.

#### Reliability of content analysis

Percent agreement between the three raters varied between 81 (comparison at the TDF level) and 100% (comparison of at the COM-B level).

## Discussion

This study used the COM-B model and TDF framework to guide the identification of barriers to and enablers of the use of an ICF-based intake tool in routine clinical oto-audiological practice as perceived by HHPs and patients. During focus groups and individual interviews, HHPs and patients reflected on factors related to their capabilities, their motivation and their physical and social opportunities to use the ICF-based intake tool. Barriers reported by HHPs were linked to a lack of knowledge and skills, time constraints, professional role and identity, and beliefs about the potential consequences of the ICF-based intake tool. Many identified enablers related to the environmental context. Patients were generally willing to use the ICF-based intake tool but reported some barriers with regard to beliefs about potential negative consequences of the tool (e.g., loss of personal contact with the HHP and compromised conversations with the HHP). The most relevant HHP- and patient specific barriers and enablers are discussed below.

### Hearing health professionals

HHPs expressed a number of advantages of using the intake tool and preferences over current practice (i.e., motivational enablers). One advantage was the potential benefit that patients could be better prepared for the intake (e.g., patients may become better aware of and specify their actual range of complaints). Also valued was the potential benefit for the HHP that the tool could help obtain a more complete picture of the patient, and could serve to manage patient expectations about the treatment and to manage patient complaints. These expectations are in accordance with the tool’s aims as described in the Introduction and these enablers should therefore be taken into account when implementing the tool.

A large number of barriers identified for the HHPs were also identified in previous studies on using PROMs in clinical practice [19, 23, 30, 42, 43]. These include perceived lack of time to use the tool and additional burden on HHPs; skepticism regarding the usefulness of the tool and its advantage as compared to current practice; the benefit to patient care is perceived to be only theoretical; and the risk that the tool might replace the patient-doctor interaction. Concerns were also raised about to the content of the ICF-based intake tool, i.e., that the items in the tool assess factors the HHP is not familiar with and/or feels incapable of handling (e.g., psychosocial aspects). Other concerns regarded the suitability of the tool for all groups of patients (in terms of diagnosis group). These concerns may lead to behavior that can hamper the targeted behavior change and therefore implementation. It should be noted that these concerns were strongest among the ENT surgeons and less among the audiologists. Analysis using the TDF suggested that HHPs’ capabilities could benefit from enhancing their knowledge about and skills to incorporate the bio-psychosocial approach of the ICF, as well their beliefs about their capabilities, goals in their intakes, and beliefs regarding the consequences of using the tool (motivation).

As mentioned previously, our aim is to develop an intake tool that is viable in all patients who visit an AC or ENT practice with any ear complaint. For this purpose, the CSHL was used as a reference, although some additions were made to render the tool suitable for all types of patients. However, HHPs questioned whether the tool was relevant and necessary for all of their patient groups, as they felt some ear and hearing problems require very straightforward and evident treatment (e.g., eardrops for a simple ear infection). From the perspective of the CSHL this is a biomedical view, which is contrary to the comprehensive functioning view that is implementation of the CSHL into practice aims for. Moreover, the HHPs’ skepticism about the redundancy of the tool for some patient groups was in strong contrast with the patient findings. All participating patients (see Table 2) saw relevance in the tool, including patients with ‘well-defined’ ear and hearing problems and ‘evident’ treatment options. It should be noted that the broad applicability of the tool still has to be demonstrated in practice. The HHPs’ concern will be addressed during the field-test study of the tool and possible adjustments will be made before final implementation into routine practice.

HPPs (particularly ENT surgeons) also reported that the tool did not align fully with their professional identity and norms. Dealing with perceptions of compatibility of new tools and interventions with existing norms is known to be challenging (e.g., [21, 44]). An intervention is needed to motivate HHPs and reassure them that the use of PROMs is potentially beneficial and can accommodate their professional identity, which ultimately leads to improved quality of care. Hanbury (2017) recommends the use of the following strategy: emphasize the commonality between PROMs and current ways of working [21]. She suggests that promoting PROMs by drawing attention to how PROMs are just another way of gaining information to inform decision making (rather than imposing a new way of working) may facilitate implementation.

The HHPs highlighted several conditions in the environmental context that could lead to a potentially successful use of the intake tool. These enablers related to the design of the tool (including the preference for a patient-administered, digital tool), and (digital) environmental structures (i.e., integration in the Electronic Patient File (EPF)). Migration of PROM to the EPF system has been shown to be feasible in other studies but requires local engagement [45, 46]. Software for an eHealth-PROM should ensure that the tool provides all desired functionalities and can accommodate possible future changes [47]. Furthermore, HHPs reported that is important for them to be able to interpret the scores immediately, which has been recommended by other studies as well [13]. In our study, prompts were identified as possibly important strategies to simplify the use of the intake tool in routine daily practice, including prompts for referral pathways for problems that are perceived as being outside the HHPs’ expertise. Developing strategies that guide HHPs to act on patient problem areas that they deem vague or outside their area of expertise is reported in the literature [13]. The ISOQOL guideline for the implementation of PROMs in clinical practice describes three solutions for this: 1) utilization of disease management pathways (i.e., prompting a specific action for follow-up), 2) further exploration of patients’ problems identified by the PROM to gain full understanding of the problem(s) and 3) utilization of multiple team members to address complex patient problems. Another enabling factor mentioned by the HHPs was that the tool should only show the responses of items that indicate a problem. However, such a method can only be applied when valid cut-off points are available. This should be taken into account in the future tool.

To enhance strategies for responding to issues identified by PROMs, Snyder et al. (2012) state that it is essential to train clinicians in how to interpret scores and how to respond to the identified patients’ problems before implementing PROMs [48]. Generating standard operating procedures can ensure consistency in adopting a new approach as the new norm in health care practice [49]. However, the HHPs participating in the current study expressed the concern that the tool would cause an overly standardized way of performing the intake (‘a fixed framework’). Our results therefore confirm the recommendations by the National Institutes of Health (NIH) that this standardization must be balanced with the need for flexibility in integrating the ICF-based intake tool in the clinical workflow, in order to limit perceived burden of the HHPs [30].

Lack of time was perceived as an important HHP barrier to implementation of the intake tool. Time is also a frequently mentioned obstacle to implementing PROMs [30], which is also consistent with previous observations that clinicians are often of the opinion that a change in clinical practice will automatically be accompanied by an increase in workload [25]. However, research shows that this is not necessarily the case. The study by Engelen et al. (2011) showed that adding feedback of health-related quality of life via PROMs did not lengthen consultation duration [50]. Another study suggested that the barrier ‘limited time’ is raised because of the idea that time has to be spent on tasks that are perceived as not supporting the professional’s role, rather than time being regarded as a limited resource in itself [44].

Possible ways to change HHP behavior can be found in (PROM-) implementation literature. For example, Michie and colleagues (2005) recommended using persuasive communication, providing information regarding the link between target behavior (here: using the intake tool) and outcome (here: anticipated patient benefit, patient-centeredness, valued based health care), and targeting barriers relating to knowledge and perceptions of the consequences of adopting the new behavior/way of working by providing feedback [51]. The latter is supported by a systematic review of facilitators and barriers to implementing PROMs in clinical palliative care practice, which demonstrates that providing feedback to clinicians can be a powerful tool to influence beliefs and attitudes towards to use of PROMs in clinical practice [43]. In a next study such intervention components will be further explored and developed.

#### Differences between ENT surgeons and audiologists

The larger range of barriers mentioned by the ENT surgeons as compared to the audiologists, suggests that ENT surgeons were most critical about the tool. The interviewed audiologists seemed to be more willing to apply the intake tool into practice; they acknowledged its potential value to construct a complete picture of the patient, and to not overlook important patient problems. The difference in the extent to which the ICF categories of the Brief CSHL overlapped with current practices of audiology and ENT was shown in our previous study [9]. The audiology patient intake documentation covered the bio-psychosocial categories of the Brief CSHL to a much higher degree (i.e., 81%) than the intake documentation of the otology patients (i.e., 63%). It should be noted that the audiologists participating in this study were all used to working in a multidisciplinary setting and many of them were familiar with the ICF. It is therefore likely that the concepts of the ICF were already partly integrated in their way of clinical thinking and their current audiology practice. The ENT surgeons’ stronger focus on biomedical aspects may also be explained by the fact that they see many patients with problems relating to the structure of the ear, for which a structural treatment is possible (e.g., ossicular chain reconstruction operations). This focus may cause less time and attention to be spent on psychosocial and contextual factors. Also, as mentioned earlier, audiologists already apply different PROMs in their current intake practice and appreciate their value in adding important information to the intake process. In current otology intake practice no PROMs are applied for clinical use. The ENT surgeon- and audiologist-specific findings have potential implications for the implementation of the intake tool: perhaps discipline-specific implementation interventions should be considered.

### Patients

Patients were generally positive and willing to use the tool. Patients’ motivation to use the intake tool seemed to be especially enhanced by the enablers that related to the perceived benefits of the tool’s goals and patient’s beliefs about the positive consequences of the tool. Perceived benefits were focused on an increased patient engagement in care, with the intake tool facilitating better preparation for the intake visit with the HHP and more focus on their specific complaints and needs. The patients’ positive response to using the intake tool is consistent with the increased willingness of patients to share their data with clinicians [52, 53].

Despite these predominantly positive perceptions, concerns were also expressed, specifically regarding the loss of personal contact with the HHP and compromised conversations with the HHP. This is a common perceived belief, which needs to be anticipated in the text introducing the tool to patients [47]. An important aim of the tool is that the provided information adds to the patient-HHP conversation and does not limit the discussion about possible causes and consequences. The intake tool’s aim is to provide a complete picture of the patient’s complaints and needs, facilitating personal attention of and conversation with the HHP and thereby serving as a communication tool that ultimately leads to an agreed-upon treatment approach.

The primary reported patient barrier to the use of PROMs in the literature is perceived burden. This means that the tool should not be too long, should be easy to use and should have clinical impact [30]. In addition it is argued that if PROM-reports automatically trigger events that mitigate the problem (e.g., communication with the doctor, patient education), the perception of burden is mitigated as well, and patients are more willing to accept the time and effort required to answer questions [30]. The recommended amount of time for any PROM is 10–15 minutes [18], which was supported by our findings as well. Also, a critical driver of high patient compliance with PROMs in other studies is that patients know their questionnaire responses are reviewed by the doctor and used in the clinical consultation [46]. This emerged from our findings as well. Moreover, this is consistent with anticipated patient expectations HHPs mentioned in the focus groups. However, HHPs reported this as a barrier to using the intake tool, as they feared the responsibility of having to review and act upon all patient responses. This contradiction requires careful consideration in the implementation plan.

Some patients expressed a preference for an open question to add narrative comments about their specific complaints/reason for visit. This is similar to findings of another qualitative study of patient and clinician views on QoL assessment in oncology practice [23], which stated that such findings help to bridge the gap between standard measurement and issues that matter to patients and should therefore be considered when implementing PROMs in clinical practice. However, our results also showed that HHP perceived the use of open questions as a barrier. Adoption of open questions should therefore be carefully considered.

Regarding the environmental opportunities and the presentation of the intake tool, the patients’ preferences, such as electronic administration at home, should be addressed in the implementation plan. In addition, there should be a back-up system for administration in the clinic (e.g., distribution of iPads to the collect data). Patients showed interest in the use of electronic portals; these have been suggested to benefit feelings of being (better) prepared for clinical appointments, higher satisfaction with treatment choices, and better adherence to medical advice [47]. However, our findings also revealed that efforts need to be made to include patients who are less likely to engage with electronic assessments (e.g., due to unfamiliarity or no access to a personal computer). Providing feedback following completion of a questionnaire is another enabler that is reported in literature and confirmed in our data. It helps patients understand the goals and motivates them to complete questionnaires again [12, 13]. It should be noted that in our study patients had difficulty indicating in what format they would prefer this feedback. We did not provide concrete visual examples of possible output options, which may have limited the range of potential options that patients came up with.

#### Overlap in barriers and enablers perceived by HHPs and patients

The observed overlap in enablers and barriers perceived by HHPs and patients is an important finding, as this will facilitate the acceptance of the intake tool. Especially with regard to the administration and design of the tool patients and clinicians seemed to be in agreement. An accepted method of PROM data collection within the clinical workflow is essential for successful implementation [54]. The concern raised by both the HHPs and patients that the intake tool could compromise the intake suggests that a PROM could be detrimental to the initial aim of promoting patient-centered care. This indicates that simply implementing PROMs in practice does not automatically result in patient-centered care, and emphasizes the importance of studying the intake tool’s implementation context. This study is an important first step, and the processes that support or impede the utilization of the intake tool should be continuously monitored in the further implementation process.

### Strengths and limitations

The strengths of the study relate to the use of the COM-B model and TDF. This approach provides an opportunity to design a theoretically informed (implementation) intervention [28]. In qualitative research, trustworthiness is highly important and should be guaranteed [41]. Trustworthiness comprises credibility (quality of the methodology used to conduct and evaluate a study), transferability (study provides rich contextual information), and dependability (consistency in the treatment of data is obtained and kept transparent). By using a theoretical approach; data from HHPs and patients; by providing quotes; and by using structured analysis, including a reliability analysis, we feel that we have ensured the trustworthiness of the study. In the implementation literature we find the recommendation that users of the potential intervention should be involved in all steps of the development and implementation of the intervention. This study included both user groups: HHPs and patients. Another strength is the inclusion of HHPs from both academic and secondary settings, enabling the broad examination of the perspectives and attitudes of Dutch oto-audiology care professionals.

Some limitations need to be discussed as well. Firstly, following the application of the behavioral change theory of COM-B in our study, we used a deductive analysis approach to identify and classify the barriers and enablers. One drawback of this approach is that it may have limited the scope and the depth of data interpretation. Because of practical reasons, data collection among HHPs was not based on data saturation principles. This may have limited the identification of other barriers and enablers to using the intake tool. However, there was considerable overlap in the responses in the discipline specific meetings, suggesting that the lack of data saturation may be limited. By including only the HHP- and the patient perspectives (i.e., the users of the intake tool), other barriers or enablers of importance may have been overlooked. The wider health care system include a broader range of factors that may affect the successful implementation of the intake tool. Examples concern stakeholders involved in the practical organization and ongoing support for collecting and integrating PROM data in patient records and in the clinical workflow. Moreover, the context-specific setting may limit the generalizability of our findings to some extent. The study was conducted in the Netherlands, and the transferability of the findings beyond the context of the Dutch health care system will require adaptation to the local context. Another limitation relates to the publication of the current project. This study is part of a larger overarching project that focusses on the development and implementation of the ICF-based intake tool, and publishing the studies in separate papers may reduce the clinical impact of this work [55]. However, the studies will also be presented as a consolidated package as part of a PhD thesis, which will include the overall clinical implications of the work. In addition, we feel that by publishing the research articles of this project separately, another relevant purpose is served: providing a detailed example on how to carefully apply the COM-B model to design an intervention. If all studies would have been presented in one paper, many helpful details in this regard would have been lost. Note that the publication and dissemination of this work is not sufficient for clinical implementation, as recently highlighted by Boisvert et al. (2017). They suggested that the current ‘research-to-practice pathways’, including peer-reviewed publications, may not be sufficient for an effective clinical implementation of evidence-based practice and patient-centered care in the field of audiology. Working together with the clinical work field was found to be key to ensure that clinicians and other stakeholders are integrated in the research process [56]. In the current project, staff members of the audiology and otology departments are part of the project group. The current study shows that other clinicians were also included in the development process of the tool, and the enablers and barriers they perceived will be used to develop an adequate implementation intervention.

### Implications of the study for research practice and policy

Results from this study are required to inform the development of an implementation plan aimed at incorporating the ICF based intake tool in routine clinical otology and audiology practice. Regarding the development of strategies for responding to issues identified by the ICF-based intake tool in order to facilitate implementation, additional research is required into existing possible effective treatment options and referral paths that correspond with ‘problem’ areas of functioning. Further research will also have to show whether the ICF-based intake tool is suitable and relevant for all patients visiting the audiology clinic and ENT practice. Although the rationale for using the intake tool in both audiology and otology has been outlined in the methods, the intake tool may not be suitable for all otologic patients. The optimization of the intake tool will be an ongoing process, requiring continuous evaluations, if necessary followed by modification.

## Conclusion and next steps

We aim to develop and implement an ICF-based intake tool for use in routine Dutch oto-audiology practice. This study identified barriers to and enablers of the use of the tool as perceived by HHPs and patients based on the COM-B and TDF. For the implementation to succeed, HHPs’ knowledge, skills and motivation regarding the relevance, clinical usefulness and clinical benefit of the tool need to be enhanced. Patients motivation to use the tool can be enhanced by providing clear and specific information on its purpose and relevance. For both HHPs and patients, opportunities in the environmental context and resources provide additional targets for successful implementation. This qualitative work is a pre-implementation step. In a next step, strategies for the implementation of the ICF-based intake tool will be developed based on the barriers and enablers that were identified in the current study. In addition, evidence on interventions from other implementation studies will be used. The final implementation intervention will be determined via a consensus procedure with relevant stakeholders.

## Supporting information

S1 Appendix COM-B components and their related TDF domains, definitions and theoretical constructs. (DOCX)

S2 Appendix Interview guides used in the focus groups and individual interviews. (DOCX)
