# Supplementary material for: Barriers to and enablers of the implementation of an ICF-based intake tool in clinical otology and audiology practice—A qualitative pre-implementation study
Source: PLoS One. 2018 Dec 11;13(12):e0208797. doi: 10.1371/journal.pone.0208797 (PMC6289452; doi:10.1371/journal.pone.0208797)
Supplement: S1 Appendix — (DOCX) [file pone.0208797.s001.docx]

**S1 Appendix: COM-B components and their related TDF domains, definitions and theoretical constructs**^[[1]](#footnote-1)^

| **COM-B component** (definition) | **TDF domain** (definition, *theoretical constructs*) |
| --- | --- |
| **Physical capability**  Physical skill, strength or stamina | **Physical skills** |
| **Psychological capability**  Knowledge or psychological skills, strength or stamina to engage in necessary mental processes | **Knowledge**  An awareness of the existence of something.  *Knowledge (including knowledge of condition/ scientific rationale); procedural knowledge; knowledge of task environment.* |
|  | **Cognitive and interpersonal skills**  An ability or proficiency acquired through practice.  *Skills; skill development; competence; ability; interpersonal skills; practice; skill assessment* |
|  | **Memory, attention, and decision processes**  The ability to retain information, focus selectively on aspects of the environment and choose between two or more alternatives.  *Memory; attention; attention control; decision making; cognitive overload/ tiredness* |
|  | **Behavioral regulation**  Anything aimed at managing or changing objectively observed or measured actions.  *Self-monitoring; breaking habit; action planning* |
| **Physical opportunity**  Opportunity afforded by the environment involving time, resources, locations, cues, physical ‘affordance’ | **Environmental context and resources**  Any circumstance of a person’s situation or environment that encourages the development of skills and abilities, independence, and adaptive behavior.  *Environmental stressors; resources/ material resources; organizational culture/ climate; salient events/ critical incidents; person x environment interaction; barriers and facilitators* |
| **Social opportunity**  Opportunity afforded by interpersonal influences, social cues and cultural norms that influence the way that we think about things, e.g., the words and concepts that make up our language | **Social influences**  Those interpersonal processes that can cause individuals to change their thoughts, feelings, or behaviors  *Social pressure; social norms; group conformity; social comparisons; group norms; social support; power; intergroup conflict; alienation; group identity; modelling* |

| **Reflective motivation**  Reflective processes involving plans (self-conscious intentions) and evaluations (beliefs about what is good and bad) | **Social/professional role and identity**  A coherent set of behaviors and displayed personal qualities of an individual in a social or work setting.  *Professional identity; professional role; social identity; identity; professional boundaries; professional confidence; group identity; leadership; organizational commitment* |
| --- | --- |
|  | **Beliefs about capabilities**  Acceptance of the truth, reality, or validity about an ability, talent , or facility that a person can put to constructive use.  *Self-confidence; perceived competence; self-efficacy; perceived behavioral control; beliefs; self-esteem; empowerment; professional confidence* |
|  | **Optimism**  The confidence that things will happen for the best or that desired goals will be attained.  *Optimism; pessimism; unrealistic optimism; identity* |
|  | **Intentions**  A conscious decision to perform a behavior or a resolve to act in a certain way.  *Stability of intentions; stages of change model; trans theoretical model and stages of change* |
|  | **Goals**  Mental representations of outcomes or end states that an individual wants to achieve.  *Goals (distal/ proximal); goal priority; goal/ target setting; goals (autonomous/ controlled); action planning; implementation intention* |
|  | **Beliefs about consequences**  Acceptance of the truth, reality, or validity about outcomes of a behavior in a given situation.  *Beliefs; outcome expectancies; characteristics of outcome expectancies; anticipated regret; consequences* |
| **Automatic motivation**  Automatic processes involving emotional reactions, desires (wants and needs), impulses, inhibitions, drive states and reflex responses | **Reinforcement**  Increasing the probability of a response by arranging a dependent relationship, or contingency, between the response and a given stimulus.  *Rewards (proximal/ distal, valued/ not valued, probable/ improbable); incentives; punishment; consequences; reinforcement; contingencies; sanctions* |
|  | **Emotion**  A complex reaction pattern, involving experiential, behavioral, and physiological elements, by which the individual attempts to deal with a personally significant matter or event.  *Fear; anxiety; affect; stress; depression; positive/ negative affect; burn-out* |

1. *adopted from Michie S, Atkins L, West R (2014). The behavior change wheel: a guide to designing interventions (1^st^ ed.). Silverback Publishing: London.*  [↑](#footnote-ref-1)
