# Supplementary material for: Barriers to and enablers of the implementation of an ICF-based intake tool in clinical otology and audiology practice—A qualitative pre-implementation study
Source: PLoS One. 2018 Dec 11;13(12):e0208797. doi: 10.1371/journal.pone.0208797 (PMC6289452; doi:10.1371/journal.pone.0208797)
Supplement: S2 Appendix — (DOCX) [file pone.0208797.s002.docx]

**S2 Appendix: Interview guides used in the focus groups and individual interviews**

***Hearing health professionals : Topic guide used in the focus groups and in one of the individual interviews (one audiologist).***

1. Can you describe the intake process in your current practice?
2. In an ideal situation, what should the intake consultation look like in your view?
3. (How) Could a different or new method support you in this ideal situation?
4. What requirements should this method meet?
5. Suppose patients would answer questions about a number of topics that related to their functioning with their ear/hearing problems in their daily lives, and you would receive the answers to these questions in an overview. And you would review these answers some time before or during the intake appointment. What would you think of such a method?
6. What requirements should this method meet such that you would want to use the method?
   1. What knowledge would you need to have in order to be able to use the method?
   2. Try to think very practically: What should happen in practice for you to use the method? How should the environment be adapted for you to use the method?
   3. What would motivate you to use the method?

***Patients: Structured interview questions used in the individual interviews***

1. Try to think of an earlier intake conversation that you had. It can be with *any* health care professional. What was your experience back then? What did you like and what did you not like about this intake conversation? (possible follow-up question: What would you like to see improved in the intake conversation?)
2. What topics do you hope that a healthcare provider would ask about/ would have specific attention for?
3. In an ideal situation, what should an intake consultation look like in your view?
4. In this ideal situation, would an overview of the relevant topics that you just mentioned, which you can prepare in advance, support you in any way?
5. What requirements should this method meet?
6. Suppose we shape the method in such a way that you as a patient would complete questions about relevant topics prior to the intake consultation, and you would discuss the summary of your answers during the intake conversation with the ENT-doctor or audiologist. What do you think of such a form/method?
7. Do you think it is important to be provided with information about why it would be important for you to fill in the questionnaire?
8. What would you like to know about using the questionnaire?
   1. Knowledge about relevance?
   2. Knowledge about how to fill in the questionnaire? (instructions)
9. Try to think very practically. What should happen in practice for you to fill in the questionnaire?; How should the environment be adapted to fill in the questionnaire?
   1. What is the maximum time that you would be prepared to spend on filling out the questionnaire?
   2. Where and in what way would you like to receive the questionnaire (at home/ in the waiting room, via the internet / on paper)?
   3. In what way would you want to obtain the (overview of your) results or answers?
   4. Would you find it important to know whether or not other patients filled in the questionnaire as well?
   5. Would you like to receive reminders for completing the questionnaire in time?
   6. Would you want any support or help from others, like your partner or caregiver, to fill in the questionnaire?
10. What would motivate you to fill in the questionnaire?
    1. Do you want to feel a sense of pleasure (fun) or satisfaction during or after completing the questionnaire?
    2. Would you like to feel that it is relevant to fill in the questionnaire? For example, that the negative consequences outweigh the positive consequences of filling in the questionnaire?
